# Hot-Water Baths in Puerperal Peritonitis

**Published:** 1886-08

**Authors:** F. W. Fitz-Gerald

**Affiliations:** 318 Centre avenue


### DOMESTIC CORRESPONDENCE.

---

### HOT-WATER BATHS IN PUERPERAL PERITONITIS.

TO THE EDITORS OF THE *Chicago Medical Journal and Examiner.*

*Gentlemen:*

In February, 1874, Mrs. F. was attended by two physicians, for what was pronounced to be peritonitis, for three weeks, she growing worse steadily. Her condition at that time was as follows: High fever, feeble pulse, 130, tympanitis so pronounced that a regular arch was formed from the neck to the pubes, severe pain and inability to swallow even a tablespoonful of fluid; urine had to be drawn with catheter. In that state the prognosis was bad, and the physicians agreed that she could not live twenty-four hours longer.

I then commenced to immerse the patient in hot water raised to the temperature of 150°, and kept her in the bath at that degree of heat for an hour, and continued to do so three times a day for the first four days, then twice a day for the remainder of the week, after which I used the hip bath once a day.

When first put into the bath she was so weak and the tympanitis so pronounced, I could not keep her head from sinking under the water while the body floated. I had recourse to a heavy log surmounted by a pillow, on which I rested her chin, she being face down in order to facilitate the escape of gas *per anum*, which took place in about half an hour after immersion. It was most offensive, and the room had to be disinfected and ventilated to prevent her from vomiting. The gas continued to escape in large quantity during each immersion. The benefit derived from the first bath was so marked that for the first time in three weeks she slept soundly for at least four hours, and on awakening could take some burned brandy. She gradually gained, and on the third day asked for the bedpan, under the impression that her bowels were about to be moved. On removing the pan I was surprised to find from a pint to a pint and a half of pure laudable pus instead of fæces. The discharge of pus continued for about a week, when it ceased entirely. She made an excellent recovery, no fistula being discoverable, and in the month of June traveled from Laramie City, Wyoming, to St. Louis. She is still living and enjoying tolerably good health. Yours, etc.,

F. W. FITZ-GERALD, M. D.

318 Centre avenue.
July 14, 1886.

